# Deep Learning Approach for Negation and Speculation Detection for Automated Important Finding Flagging and Extraction in Radiology Report: Internal Validation and Technique Comparison Study

**DOI:** 10.2196/46348

**Published:** 2023-04-25

**Authors:** Kung-Hsun Weng, Chung-Feng Liu, Chia-Jung Chen

**Affiliations:** 1 Department of Medical Imaging Chi Mei Medical Center, Chiali Tainan Taiwan; 2 Department of Medical Research Chi Mei Medical Center Tainan Taiwan; 3 Department of Information Systems Chi Mei Medical Center Tainan Taiwan

**Keywords:** radiology report, natural language processing, negation, deep learning, transfer learning, supervised learning, validation study, Bidirectional Encoder Representations from Transformers, BERT, clinical application, radiology

## Abstract

**Background:**

Negation and speculation unrelated to abnormal findings can lead to false-positive alarms for automatic radiology report highlighting or flagging by laboratory information systems.

**Objective:**

This internal validation study evaluated the performance of natural language processing methods (NegEx, NegBio, NegBERT, and transformers).

**Methods:**

We annotated all negative and speculative statements unrelated to abnormal findings in reports. In experiment 1, we fine-tuned several transformer models (ALBERT [A Lite Bidirectional Encoder Representations from Transformers], BERT [Bidirectional Encoder Representations from Transformers], DeBERTa [Decoding-Enhanced BERT With Disentangled Attention], DistilBERT [Distilled version of BERT], ELECTRA [Efficiently Learning an Encoder That Classifies Token Replacements Accurately], ERNIE [Enhanced Representation through Knowledge Integration], RoBERTa [Robustly Optimized BERT Pretraining Approach], SpanBERT, and XLNet) and compared their performance using precision, recall, accuracy, and *F*_1_-scores. In experiment 2, we compared the best model from experiment 1 with 3 established negation and speculation-detection algorithms (NegEx, NegBio, and NegBERT).

**Results:**

Our study collected 6000 radiology reports from 3 branches of the Chi Mei Hospital, covering multiple imaging modalities and body parts. A total of 15.01% (105,755/704,512) of words and 39.45% (4529/11,480) of important diagnostic keywords occurred in negative or speculative statements unrelated to abnormal findings. In experiment 1, all models achieved an accuracy of >0.98 and *F*_1_-score of >0.90 on the test data set. ALBERT exhibited the best performance (accuracy=0.991; *F*_1_-score=0.958). In experiment 2, ALBERT outperformed the optimized NegEx, NegBio, and NegBERT methods in terms of overall performance (accuracy=0.996; *F*_1_-score=0.991), in the prediction of whether diagnostic keywords occur in speculative statements unrelated to abnormal findings, and in the improvement of the performance of keyword extraction (accuracy=0.996; *F*_1_-score=0.997).

**Conclusions:**

The ALBERT deep learning method showed the best performance. Our results represent a significant advancement in the clinical applications of computer-aided notification systems.

## Introduction

### Background

Timely and effective communication of test results is essential in modern medicine. To promptly address patients’ problems, hospitals must ensure that the test results are completed without delay and that clinicians are aware of substantial abnormal findings. Delayed or failed communication of important findings by the department performing the test and the clinical team can increase the risk of adverse patient events and result in medical malpractice and compensation, especially for potentially life-threatening and important diagnoses [1].

Although radiology reports are the primary method of communication between radiology and clinical departments, the fact that a radiologist produces a report does not necessarily mean that the clinician reads it entirely. Ignácio et al [2] showed that only 55.7% of clinicians read the entire report thoroughly. Reda et al [3] showed that >40% of clinicians read only the conclusions or only read the conclusions in detail. More than 30% of clinicians have made preventable medical errors because they did not read radiology reports carefully. Even if the radiologist has made the correct diagnosis in the report, the clinician may still miss it.

To address these communication issues, current radiology guidelines [4] now require radiologists to go beyond report completion and use additional communication methods for reports with significant findings, including flagging or alerting the report, e-mailing, or direct verbal communication via telephone. Natural language processing can also automatically extract data from radiology reports, for example, automatically extracting important diagnoses, follow-up data, or management recommendations or automatically identifying reports that require specific action [5]. These methods can help to identify important information in radiology reports or reports that need to be read in detail to alert clinicians.

In addition, the laboratory information system (LIS) used in hospitals today can automatically highlight abnormalities found in tests and display them differently to ensure that clinicians do not miss important findings, such as using different colors or special symbols [6]. For example, in our hospital, if a patient has undergone a routine blood test and some of the blood cell counts are abnormal, the LIS will automatically display the results on the computer screen in a unique color for the abnormal values and a typical color for the others. The LIS also displays important keywords (eg, nodules) within radiology reports in different colors.

However, because most radiology reports are freely typed by radiologists in an unstructured manner, both techniques encounter challenges. Negative and speculative statements are significant problems.

Radiologists can use negative statements to communicate the absence of specific diagnoses and provide a clearer picture of the patient’s condition. For example, the statement “No definite CT evidence of aortic dissection” informs the clinician that the patient’s condition is not related to aortic dissection.

The diagnoses in the speculative statements may or may not be related to the actual abnormal findings. The radiology report may contain speculative statements in the presence of an imaging finding of uncertain significance that requires further investigation, for example, “RUL lung nodule. Lung cancer should be suspected.” In such cases, the diagnoses (lung cancer) in the speculative statements are related to abnormal findings. Even if the radiologist finds no problems with the study, the radiology report may still contain speculative statements to prevent potential medicolegal issues. Disclaimer (eg, “10%-15% of cases of breast cancer are missed on mammograms” [7]) or statement of limitations (eg, “non-enhanced images, small lesion may be obscured”) are common examples. In such cases, the diagnoses (breast cancer or lesion) in the speculative statements are unrelated to the actual diagnoses.

A notification system that does not distinguish whether diagnostic Information is contained in negative or speculative statements unrelated to abnormal findings and annotates or extracts all of them to “alert” the clinician may generate excessive false alarms. Excessive false alarms can overload the clinician’s senses and lead to the “cry wolf” phenomenon, causing alarm fatigue. Consequently, clinicians may delay detection or even ignore truly valuable alerts, posing a risk to patients, especially if the percentage of false alarms is high [8].

This study aimed to address the potential analytical inaccuracies resulting from negative and speculative statements in radiology reports and to facilitate the use of unstructured reports by hospital information systems.

### Prior Work

Current studies have adopted various approaches to detect negation and speculation, including rule-based, machine learning–based, and deep learning–based approaches [9-17].

The rule-based approach relies on experts to define the rules that are understandable to humans. NegEx, proposed by Chapman et al [18]; NegFinder, proposed by Mutalik et al [19]; NegHunter, proposed by Gindl et al [20]; and NegExpander, proposed by Aronow et al [21], are regular expression-based approaches. Regular expression-based methods have limitations, such as the inability to capture the syntactic structure and the possibility of misinterpreting the scope of the negative and speculative statements. For example, “No change of tumor” may be misinterpreted as both “No change” and “No tumor.”

Methods such as DEEPEN (Dependency Parser Negation), proposed by Mehrabi et al [22], and NegBio, proposed by Peng et al [23], analyze the syntactic structure based on grammar. These methods are more accurate than regular expression-based approaches in limiting the scope of negative and speculative statements and reducing false positives because these methods consider the dependency relationship between words. However, these methods have certain limitations. For example, errors in the analysis may occur if the grammar of the text deviates from typical norms, such as the presence of long noun phrases [23]. When analyzing text, most of these methods [18-20,22,23] split the text into sentences that are analyzed independently. The algorithms and expert-defined rules only consider a single sentence at once and do not consider both the preceding and following contexts.

With the advancement of artificial intelligence, machine learning techniques have been applied to detect negation and speculation. For example, Medlock et al [24] proposed a weakly supervised learning–based approach to predict the labels of training samples for machine learning training and used the trained models to detect speculation in biomedical texts. Rokach et al [25] compared several machine learning approaches, including the Hidden Markov Model, Conditional Random Field (CRF), decision tree, and AdaBoost, cascaded decision tree classifiers with and without the Longest Common Sequence. They found that the cascaded decision tree with the Longest Common Sequence performed best. Morante et al proposed k-nearest neighbor algorithm–based [26] and meta-learning–based approaches [27]. Ou et al [28] compared rule-based and support vector machine–based machine learning methods and obtained better performance of machine learning methods.

Later studies began investigating deep learning–based approaches and achieved better results than previous non–deep learning approaches. Qian et al [17] were the first to propose a deep learning method for negation and speculation detection using a convolutional neural network–based model by using the relative position of tokens and path features from syntactic trees as features.

By contrast, recurrent neural networks and their derivatives, such as Long Short-Term Memory (LSTM), are suitable for processing sequential data. These architectures can incorporate dependencies on preceding and following elements, making them particularly useful for natural language processing tasks, and have achieved good results in recognizing negations and speculations. For example, in a study by Fancellu et al [14], a Bidirectional LSTM (BiLSTM)–based model was applied, and it demonstrated better performance than other methods on the Sherlock data set. Lazib et al [9] compared methods, including LSTM, BiLSTM, Gated Recurrent Unit, and CRF, and showed that the recurrent neural network–based architecture performed the best. Gautam et al [15] compared several LSTM-based models and obtained the best performance using 2-layer encoders and decoders with dropouts. Taylor et al [10] applied the BiLSTM-based model to the analysis of negation in electroencephalography reports. Sergeeva et al [11] proposed an LSTM-based approach and investigated the effect of expert-provided negation cues on the detection performance of the negation scopes. Sykes et al [12] compared the methods based on BiLSTM and feedforward neural networks and rule-based methods, including pyConText, NegBio, and EdIE-R, for negation detection in radiology reports. The BiLSTM-based approach outperformed other approaches.

BERT (Bidirectional Encoder Representations from Transformers) [29], proposed by Google in 2018, is a pretrained, transformer-based model that is effective for negation detection. Khandelwal et al [16] developed NegBERT and, in another study [13], used a multitasking approach with BERT, XLNet, and RoBERTa (Robustly Optimized BERT Pretraining Approach) for negation and speculation detection, with improved results on BioScope and Simon Fraser University review data sets compared with the control methods. Zavala et al [30] proposed a system based on BiLSTM with CRF and fine-tuned BERT; evaluated the methods on English and Spanish clinical, biomedical, and review text; and showed improved performance compared with previous methods. They also found that pretrained word embedding, especially contextualized embedding, helped to understand the biomedical text.

Numerous variants of BERT have been developed to improve performance and simplify the model. ALBERT (A Lite BERT) [31] reduces the model parameters and improves the performance through parameter sharing and matrix decomposition. DistilBERT (Distilled version of BERT) [32] uses knowledge distillation to reduce the size and improve the inference speed while retaining most of the language understanding. XLNet [33] implements autoregressive training while preserving the advantages of autoencoding models and outperforms BERT on 20 tasks. RoBERTa [34] improves the training method to outperform BERT and XLNet. ERNIE (Enhanced Representation through Knowledge Integration) [35] uses an alternative masking method to outperform BERT in Chinese tasks. SpanBERT [36] extends BERT with span-based masking and an additional training objective, resulting in a better performance on span-based tasks. DeBERTa (Decoding-Enhanced BERT With Disentangled Attention) [37] improves BERT and RoBERTa with decoupled attention, improved mask encoder, and virtual adversarial training and outperforms RoBERTa-Large on the Multigenre Natural Language Inference, Stanford Question Answering Data set, and Reading Comprehension data set from examinations tasks and humans on the SuperGLUE task. ELECTRA (Efficiently Learning an Encoder That Classifies Token Replacements Accurately) [38] outperforms BERT with a new pretraining task, Replaced Token Detection, and performs similarly to RoBERTa and XLNet with one-fourth the computation.

### Contribution of This Work

This study has implications for optimizing the performance of hospital information systems in managing unstructured electronic medical records. The key findings and results of this study are as follows.

First, we found that fine-tuned general-purpose transformer models could outperform NegEx, NegBio, and NegBERT, which are explicitly designed for negation and speculation detection. We identified sources of error in the latter 3 methods and suggested potential improvements.

Second, we found that transformer, unlike NegEx and NegBio, demonstrated the ability to perform multisentence contextual analysis and further granular classification of speculative statements as related or unrelated to abnormal findings. This capability can improve information filtering in hospital information systems to eliminate nondiagnostically relevant information.

Finally, in contrast to other studies using BERT [16,39], we found that using a lightweight transformer model and learning the cues and scopes of negative and speculative sentences in a single step can perform well.

## Methods

### Ethics Approval

**Figure 1:**
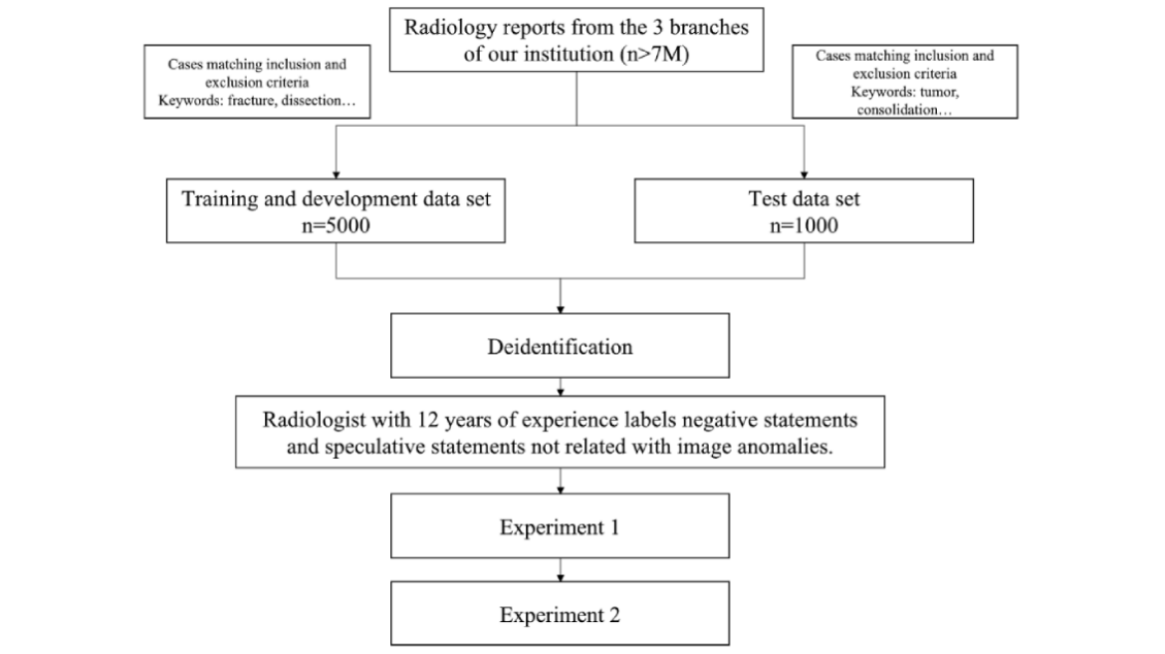
Research flow. n: number of reports.

The Chi Mei Hospital Institutional Review Board reviewed and approved this study (11105-J02). This study is a retrospective analysis study using deidentified electronic medical records, thus obviating the requirement for obtaining informed consent from the individuals. Figure 1 shows the flow diagram of the study.

### Inclusion and Exclusion Criteria

The inclusion criteria for this study were radiological examinations performed in the 3 branches of our institution between 2012 and 2022, with the reports being written in English language and the type of examination being x-ray, special radiology, computed tomography (CT), magnetic resonance imaging (MRI), or ultrasound. We included cases that met all criteria. The exclusion criteria were Chinese reports and patients aged <20 years at the time of examination. We excluded cases that met any of the exclusion criteria. Samples were collected using 2 independent keyword searches in a search engine targeting radiology reports that met the inclusion criteria but not the exclusion criteria.

### Data

#### Overview

The training and development data set consisted of 5000 radiology reports randomly selected from a keyword search using the terms “fracture,” “dissection,” “infarct,” “pneumothorax,” “extravasation,” “thrombosis,” or “pneumoperitoneum.” The test data set consisted of 1000 reports selected from a keyword search using the terms “tumor,” “consolidation,” “pulmonary TB,” “metastasis,” or “bleeding.” Keywords were selected from our institution’s list of important keywords and randomly assigned to the data sets. These keywords are referred to as “important keywords” in the study. The samples in the training and development and test data sets were mutually exclusive with no overlap.

The training and development data set was automatically partitioned into training and development data sets in a 9:1 ratio for model training. The training, development, and test data sets ratio was 9:1:2, with 4500, 500, and 1000 radiology reports, respectively.

In this study, each word or token was assigned to one of the 2 categories, as shown in Table 1: “Positive statements, or speculative statements potentially related to abnormal findings” (category 0) and “negative statements, or speculative statements not related to abnormal findings” (category 1). We combined speculative statements unrelated to abnormal findings with negative statements as a single class because of their limited representation. The rationale for category 1 is that the information conveyed is not relevant to abnormal findings and should not trigger highlights or alerts. A token is the minimum output unit of the transformer-based model’s tokenizer.

All radiology reports included in the study were deidentified by removing identifying information such as medical record number, application number, examination date, ordering department, and examination time. A radiologist with 12 years of experience (KHW) reviewed the reports and annotated all negative and speculative statements unrelated to abnormal findings using the open-source Doccano [40] software. The annotation served as the gold standard for subsequent analysis.

**Table 1:** Classification of words and tokens in this study.

Type^a^ and subtype			Example		Category^b^
Negative			Liver laceration at S6 *without active contrast extravasation*		1
**Speculative**					
	Unrelated to abnormal findings	No CT^c^ evidence of large infarct. *Suggest MRI^d^ to exclude hyperacute infarct if indicated*		1	
	Potentially related to abnormal findings	*Rt^e^ cerebellum acute infarct cannot be ruled out.*		0	
Positive			*Rt cerebellum acute infarct*		0

^a^Type refers to the type of statement.

^b^Token category in the italicized text if italicization is used. All texts without italics were classified as category 0. Category 0: positive statements or speculative statements potentially related to abnormal findings. Category 1: negative statements or speculative statements not related to abnormal findings.

^c^CT: computed tomography.

^d^MRI: magnetic resonance imaging.

^e^Rt: right.

#### Included Negations

This study included all statements in which the radiologist explicitly denied a diagnosis or a finding. Our data included morphological negation and sentential negation, which are common forms of negative statements in English text [22]. Morphological negation involves using prefixes, such as “un-” or “ir-,” to modify certain words to express negation. Sentential negation involves using negative words, such as “no” or “without,” to negate part of the statement. In addition, radiologists at the authors’ hospital often use unique symbols or abbreviations, such as “(−)” or “[−].”

#### Included Speculations

In cases where the imaging study is inconclusive but there is still the possibility of a significant abnormality, the information system should notify the clinician and allow the clinician to make the final decision. Therefore, for the task of speculation detection, our focus was limited to speculative statements that were unrelated to abnormal findings. Meanwhile, we treated speculative statements that may correlate with actual abnormal findings as equivalent to positive statements.

After reviewing the samples, we identified 2 scenarios in which speculative statements could be confidently determined to be unrelated to abnormal findings. First, the radiologist explicitly stated that there was no relevant abnormality. Second, the radiologist stated that certain diagnoses could not be evaluated owing to study limitations. In all the other scenarios, speculative statements may be associated with abnormal findings.

In the following 3 examples, we classify the diagnoses or findings written in italics as speculative statements unrelated to abnormal findings. The actual test results were normal or unrelated to these diagnoses or findings.

1. No CT evidence of pulmonary embolism. Suggest V/Q scan to exclude *small branch embolism* if indicated.
2. No CT evidence of large infarct. Suggest MRI to exclude *hyperacute infarct* if indicated.
3. *Liver tumor* cannot be excluded by noncontrast CT.

In the following 2 examples, the diagnoses or findings written in italics are speculative statements considered potentially related to actual abnormal findings:

1. Equivocal filling defect in RLL segmental pulmonary artery. Suggest V/Q scan to exclude *small branch embolism* if indicated.
2. Rt cerebellum *acute infarct* cannot be ruled out.

### Design of the Experiments

We conducted 2 experiments to evaluate the ability of general all-purpose pretrained deep learning models and existing negation and speculation-detection algorithms to identify negation and speculation in real-world radiology reports.

**Figure 2:**
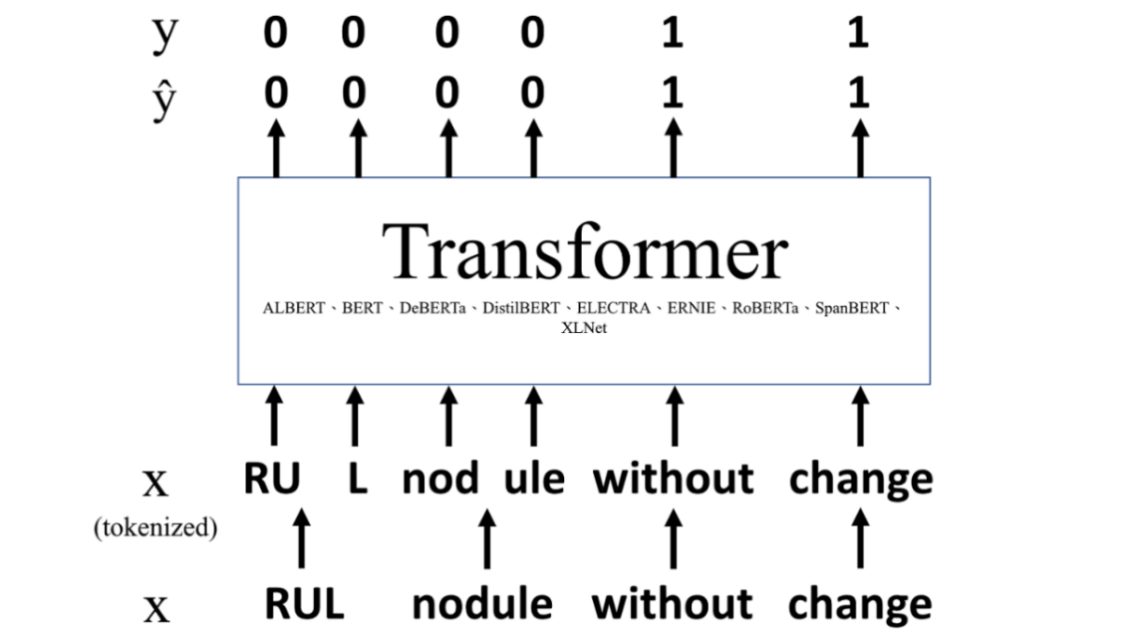
Experiment 1. X: the original text, ŷ: class predicted by the model; y: the gold standard. Category 0: positive statements or speculative statements potentially related to abnormal findings; category 1: negative statements or speculative statements unrelated to abnormal findings. ALBERT: A Lite Bidirectional Encoder Representations From Transformers; BERT: Bidirectional Encoder Representations From Transformers; DeBERTa: Decoding-Enhanced Bidirectional Encoder Representations From Transformers With Disentangled Attention; DistilBERT: Distilled version of Bidirectional Encoder Representations From Transformers; ELECTRA: Efficiently Learning an Encoder That Classifies Token Replacements Accurately; ERNIE: Enhanced Representation through Knowledge Integration; RoBERTa: Robustly Optimized Bidirectional Encoder Representations From Transformers Pretraining Approach; RUL: right upper lobe.

In experiment 1 (Figure 2), we fine-tuned several transformer-based models using our training and validation data sets. We performed token category prediction (category 0 or 1) for all tokens in the training, validation, and test data sets.

**Figure 3:**
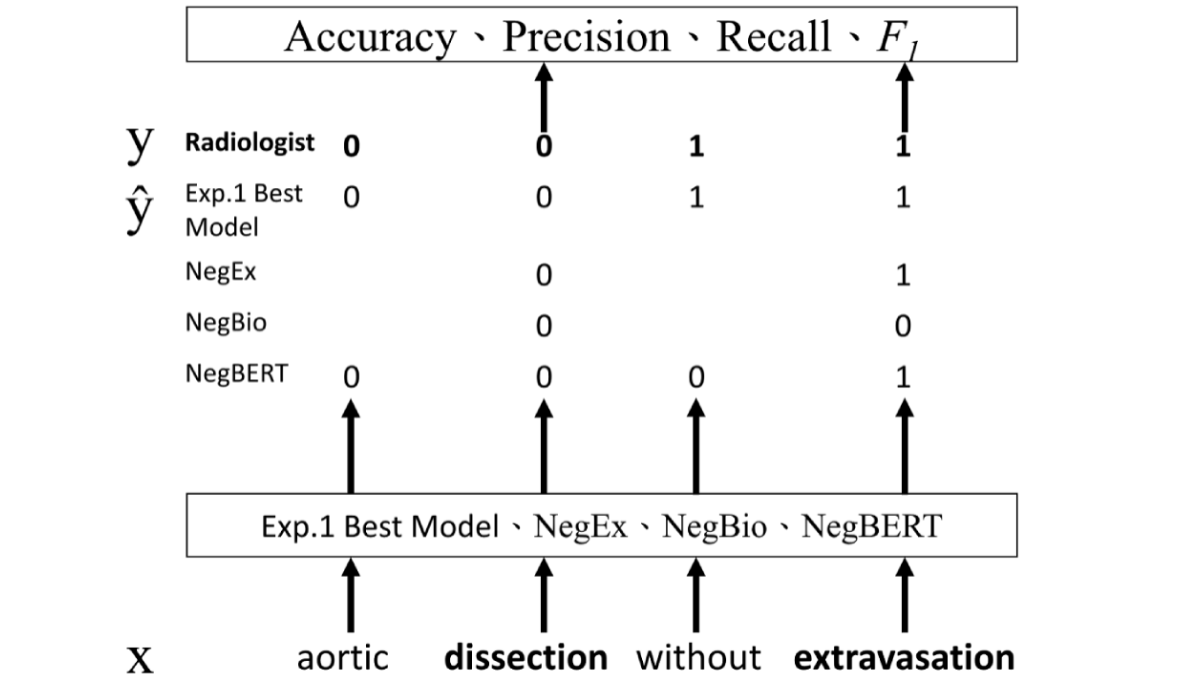
Experiment 2 Note. X: the original text; ŷ: class predicted by the model; y: the gold standard; category 0: positive statements or speculative statements potentially related to abnormal findings; category 1: negative statements or speculative statements unrelated to abnormal findings; bold text: word matching a designated “important keyword.” Exp: experiment.

In experiment 2 (Figure 3), we compared 3 negation and speculation-detection algorithms that performed well on public data sets with the best model from experiment 1. The algorithms evaluated were NegEx, NegBio, which has predefined expert rules and open-source implementation, and NegBERT, whose training code is available. We then performed category prediction (category 0 or 1) for all words that matched a given “important keyword” in the test data set. We also analyzed the sources of errors. In addition, we compared the performance of keyword extraction in positive and speculative statements potentially related to abnormal findings before and after applying various algorithms.

### Modeling in Experiments

The deep learning models used in experiment 1 were ALBERT, BERT, DeBERTa, DistilBERT, ELECTRA, ERNIE, RoBERTa, SpanBERT, and XLNet. All models were fine-tuned based on the pretrained models from Hugging Face.

We used early stopping and used the *F*_1_-score as the model evaluation metric. We used the Adam optimizer with a batch size of 16 and weight decay of 0.01. Table 2 lists the parameters of other models. We set all other unspecified parameters to the default values provided by the open-source PyTorch framework. We segmented the texts into blocks of no more than 510 characters before entering the model to avoid truncation.

We adopted a sequence-to-sequence approach for the training. The training program input the report text in the training and development data set into the model using the corresponding tokenizer and trained the model. The models predicted the token categories using the radiologist-annotated data as the gold standard. The test data set was not included in the training process.

For the NegEx algorithm, we used the negspaCy pipeline component of the open-source Spacy software [41]. The specific named entity recognition model used was “en_ner_bc5cdr_md.” In addition, we extended the recognizable entities in Spacy to include all the important keywords defined in our experiment.

We used the previously published training parameters of NegBERT, including a batch size of 8, maximum training epochs of 60, an initial learning rate of 3 × 10^−5^, and an early stopping patience of 6. We applied NegBERT for cue detection using the model “bert-base-uncased” and scope detection using the model “xlnet-base-cased.” Furthermore, we validated that the trained NegBERT showed a comparable level of performance to that reported in the original publication on the data set specified in the original study.

In addition to the configuration mentioned earlier, we made only minimal modifications to NegBio and NegBERT, such as specifying the dependent software versions, adding the necessary files to the installation, and configuring file paths to ensure the proper execution of the software.

In experiment 2, we optimized the performance of the NegEx, NegBio, and NegBERT methods. This optimization was achieved by modifying the expert-defined rules of NegEx and NegBio and using our training and development data set, as well as the negation and speculation cues we identified, to train NegBERT without using the data set from the original study.

**Table 2:** Deep learning model and training parameters used in this study.

Model	Learning rate	Warm-up steps	Adam beta1	Adam beta2	Adam epsilon	FP16^a^
ALBERT^b^	1 × 10^−5^	10,000	0.9	0.999	1 × 10^−8^	False
BERT^c^	1 × 10^−4^	10,000	0.9	0.999	1 × 10^−8^	False
DeBERTa^d^	1 × 10^−4^	10,000	0.9	0.999	1 × 10^−6^	True
DistilBERT^e^	2 × 10^−5^	0	0.9	0.999	1 × 10^−8^	False
ELECTRA^f^	1 × 10^−4^	10,000	0.9	0.999	1 × 10^−6^	False
ERNIE^g^	5 × 10^−5^	4000	0.9	0.98	1 × 10^−8^	False
RoBERTa^h^	1 × 10^−4^	10,000	0.9	0.999	1 × 10^−8^	False
SpanBERT	5 × 10^−5^	10,000	0.9	0.999	1 × 10^−8^	False
XLNet	2 × 10^−5^	10,000	0.9	0.999	1 × 10^−6^	False

^a^FP16: half-precision floating-point format.

^b^ALBERT: A Lite Bidirectional Encoder Representations From Transformers.

^c^BERT: Bidirectional Encoder Representations From Transformers.

^d^DeBERTa: Decoding-Enhanced Bidirectional Encoder Representations From Transformers With Disentangled Attention.

^e^DistilBERT: Distilled version of Bidirectional Encoder Representations from Transformers.

^f^ELECTRA: Efficiently Learning an Encoder That Classifies Token Replacements Accurately.

^g^ERNIE: Enhanced Representation through Knowledge Integration.

^h^RoBERTa: Robustly Optimized Bidirectional Encoder Representations From Transformers Pretraining Approach.

## Results

### Demographics

The data set included in this study consisted of 6000 radiology reports, including plain radiography reports (2538/6000, 42.3%), CT reports (2163/6000, 36.05%), MRI reports (668/6000, 11.13%), ultrasound reports (483/6000, 8.05%), angiography reports (97/6000, 1.62%), and reports from other types of studies (51/6000, 0.85%). The report was completed by 78 radiology residents and their attending physicians. The training, validation and test data sets were mutually exclusive with no overlap in the samples.

The data set used in this study consisted of 78,901 sentences and 704,512 words. A total of 15.01% (105,755/704,512) of all the words in the data set, were identified as negative and speculative statements unrelated to abnormal findings. Table 3 presents examples and frequencies of these statements. In this study, we defined a “word” as a contiguous sequence of one or more non–white space characters of maximum length. For example, “(−) metastasis” contains 2 words.

Of all the 16,374 cases of sentential negations identified, 15,568 (95.1%) used “no,” “without,” “not,” or “none” as the first word of the negative statement. Furthermore, of all the 2763 cases of negation using symbols or abbreviations, we observed that 2411 (87.2%) used (−), (_), ( ), or [−] at the beginning, end, or middle of the negated clause.

Table 4 presents the frequency and number of occurrences of important keywords, as defined in this study, within negative or speculative statements unrelated to abnormal findings and the total number of occurrences in the study.

**Table 3:** Types and numbers of negative and the speculative sentences unrelated to abnormal findings included in this study (N=19,467).

Type	Example	Findings, n (%)
Sentential negation	No evidence of aortic dissection	16,374 (84.11)
Symbols or abbreviations	Metastasis (−) Thrombosis: No DM^a^- HTN^b^- Anti-HCV^c^ [Negative] - lung - bone	2762 (14.19)
Speculative statements not related to abnormal findings	No CT^d^ evidence of pulmonary embolism. Suggest V/Q^e^ scan to exclude small branch embolism if indicated Metallic artifacts, lesion may be obscured	196 (1.01)
Morphological negation	This coronary CT scan is nondiagnostic.	135 (0.69)

^a^DM: diabetes mellitus.

^b^HTN: hypertension.

^c^HCV: hepatitis C virus.

^d^CT: computed tomography.

^e^V/Q: ventilation and perfusion.

**Table 4:** Occurrence and frequency of important keywords defined in this study within negative or the speculative statements unrelated to abnormal findings.

Keywords and their overall occurrences (n=11,480)	Occurrences (N+S)^a^, n (%)
Pneumothorax, n=1288 (11.22%)	976 (75.78)
Extravasation, n=182 (1.58%)	84 (46.2)
Fracture, n=2161 (18.82%)	992 (45.90)
Tumor, n=2698 (23.5%)	1025 (37.99)
Infarct, n=1364 (11.88%)	514 (37.68)
Consolidation, n=428 (3.73%)	152 (35.5)
Pneumoperitoneum, n=63 (0.55%)	19 (30)
Thrombosis, n=614 (5.35%)	143 (23.3)
Dissection, n=673 (5.86%)	147 (21.8)
Metastasis, n=1876 (16.34%)	450 (23.98)
Bleeding, n=118 (1.03%)	27 (22.9)
Pulmonary TB^b^, n=15 (0.13%)	0 (0)

^a^Number of occurrences within negative or speculative statements unrelated to abnormal findings.

^b^TB: tuberculosis.

### Result of Experiment 1

Table 5 presents the results of experiment 1. The accuracy of all transformer-based models included in this experiment was greater than 0.98 for both the training, validation, and test data sets, with macro *F*_1_-scores >0.90. The best-performing model, ALBERT, was selected for inclusion in experiment 2.

**Table 5:** Comparison of deep learning prediction performance.

	Train and validation data set					Test data set			
	Precision	Recall	*F*_1_	Accuracy	Precision		Recall	*F*_1_	Accuracy
ALBERT^a^	0.992	0.990	0.992	0.998	*0.973* ^b^		*0.943* ^b^	*0.958* ^b^	*0.991* ^b^
BERT^c^	0.980	0.986	0.983	0.995	0.960		0.930	0.945	0.989
DeBERTa^d^	0.989	0.971	0.975	0.993	0.958		0.859	0.906	0.980
DistilBERT^e^	0.994	0.990	0.992	0.998	0.980		0.912	0.945	0.988
ELECTRA^f^	0.982	0.982	0.982	0.995	0.956		0.943	0.950	0.989
ERNIE^g^	0.987	0.984	0.986	0.996	0.963		0.920	0.941	0.988
RoBERTa^h^	0.959	0.979	0.969	0.991	0.890		0.933	0.911	0.980
SpanBERT	0.992	0.992	0.992	0.998	0.958		0.932	0.945	0.988
XLNet	0.993	0.993	0.993	0.998	0.970		0.943	0.957	0.990

^a^ALBERT: A Lite Bidirectional Encoder Representations From Transformers.

^b^Italics highlight that the performance of A Lite Bidirectional Encoder Representations From Transformers is the best comparing to the control method across various performance metrics.

^c^BERT: Bidirectional Encoder Representations From Transformers.

^d^DeBERTa: Decoding-Enhanced Bidirectional Encoder Representations From Transformers With Disentangled Attention.

^e^DistilBERT: Distilled version of Bidirectional Encoder Representations from Transformers.

^f^ELECTRA: Efficiently Learning an Encoder That Classifies Token Replacements Accurately.

^g^ERNIE: Enhanced Representation through Knowledge Integration.

^h^RoBERTa: Robustly Optimized Bidirectional Encoder Representations From Transformers Pretraining Approach.

### Result of Experiment 2

Before optimization, the performance of NegBio and NegBERT was suboptimal. The *F*_1_-scores for NegEx, NegBio, and NegBERT were 0.889, 0.587, and 0.393, respectively. Our optimization significantly improved the performance of NegBio and NegBERT by increasing their *F*_1_-scores by 0.239 and 0.588, respectively.

Table 6 shows the performance of ALBERT and optimized NegEx, NegBio, and NegBERT. The precision, recall, and *F*_1_-score of our fine-tuned transformer-based model (ALBERT) were better than those of the optimized NegEx, NegBio, and NegBERT.

Table 7 shows the performance evaluation of keyword extraction before and after applying the different negation and speculation-detection algorithms. The ALBERT method resulted in the most significant performance improvement in extracting keywords from positive and speculative statements potentially associated with abnormal findings.

**Table 6:** Comparison of performance of A Lite Bidirectional Encoder Representations From Transformers (ALBERT) and optimized NegEx, NegBio, and NegBERT in the test data set.

	Precision	Recall	*F*_1_	Accuracy
ALBERT	0.*991*^a^	0.*992*^a^	0.*991*^a^	0.*996*^a^
NegEx	0.886	0.958	0.921	0.959
NegBio	0.860	0.794	0.826	0.917
NegBERT	0.992	0.970	0.981	0.991

^a^Italics highlight that the performance of ALBERT is the best comparing to the control method (NegEx, NegBio, NegBERT) across various performance metrics.

**Table 7:** Comparison of the performance of keyword extraction in the test data set both before and after applying A Lite Bidirectional Encoder Representations From Transformers (ALBERT) and optimized NegEx, NegBio, and NegBERT.

	Precision	Recall	*F*_1_	Accuracy
ALBERT	0.*998*^a^	0.*997*^a^	0.*997*^a^	0.*996*^a^
NegEx	0.986	0.959	0.972	0.959
NegBio	0.934	0.958	0.945	0.917
NegBERT	0.99	0.998	0.994	0.991
Baseline^b^	0.752	1.00	0.859	0.752

^a^Italics highlight that the performance of ALBERT is the best comparing to the control method (NegEx, NegBio, NegBERT) and baseline (no negation or speculation detection were performed) across various performance metrics.

^b^All named entities considered “positive.” No negation or speculation-detection algorithm was applied.

### Sources of Errors

#### Overview

We analyzed the sources of the errors (Table 8). Despite changes in the rules defined by the experts, errors persisted in NegEx and NegBio. We identified the following causes:

**Table 8:** Analysis of the causes of errors in different methods (after optimization).

Method and cause of the wrong prediction^a^			Counts, n (%)
**NegBio (n=177)**			
	Errors in the extraction of named entities	58 (32.8)	
	Symbol-related errors	49 (27.7)	
	Tokenization error	21 (11.9)	
	Errors in the prediction of speculative statements	14 (7.9)	
**NegEx (n=87)**			
	False-positive prediction related to speculative statements	37 (42)	
	Trigger word not triggered	21 (24)	
	Incorrect scope resolution	16 (18)	
	Symbol-related errors	6 (6)	
**NegBERT (n=20)**			
	All false-negative predictions	16 (80)	
	All false-positive predictions	4 (20)	
	False-positive predictions related to speculative statements unrelated to abnormal findings	0 (0)	
**ALBERT^b^ (n=9)**			
	All false-positive predictions	5 (55)	
	False-positive predictions related to speculative statements unrelated to abnormal findings	0 (0)	

^a^The table only list the most important causes of identifiable error.

^b^ALBERT: A Lite Bidirectional Encoder Representations From Transformers.

#### Findings of NegEx

First, we found many errors owing to incompatibility between the NegEx method for identifying speculative statements and the study requirements. NegEx made identical predictions for all keywords in the identified speculative statements regardless of their relevance to abnormal findings. However, our study categorized keywords in speculative sentences differently based on their relevance to abnormal findings, leading to discrepancies with NegEx’s results.

Second, the trigger word would only sometimes trigger. For example, in the phrase “1.No evidence of tumor,” the trigger word “No” would not be recognized because it was concatenated with the character “1.” without any intervening space.

Third, errors also occurred owing to the misinterpretation of the scope of negation and speculation, such as misinterpreting “No improvement of the tumor” as “No tumor.”

Fourth, errors occurred in the presence of symbols in radiology reports; for example, the use of special symbols by radiologists that are undefined in the trigger word or the confusion caused by the co-occurrence of special symbols that express a positive and a negative statement: (−) fatty liver and (+) portal vein thrombosis.

#### Findings of NegBio

We identified the following errors when using NegBio:

First, errors occurred in named entity extraction. The named entities in NegBio’s output file might be missing target keywords or had incorrect positions, resulting in incorrect future analyses.

Second, errors occurred when the radiology report contained negations using symbols or abbreviations, such as “metastasis (−).” Our analysis showed that these symbols could lead to unpredictable results in syntactic structure analysis and subsequent analyses.

Third, combining words with numerals or punctuation marks leads to errors in tokenization and subsequent analysis. For example, “1.No” in “1.No obvious acute infarct or brain metastasis” was not correctly parsed as “No.”

Fourth, many errors occurred because NegBio made identical predictions for diagnostic keywords in all speculative sentences, regardless of their relevance to abnormal findings. This behavior was inconsistent with the labeling of this experiment.

#### Findings of NegBERT and ALBERT

We observed the suboptimal performance of NegBERT when applied to corpora from different domains and tasks. The performance of NegBERT trained on the Simon Fraser University review corpus was suboptimal when evaluated on our corpus and task. Retraining NegBERT with our data significantly improved its performance, indicating that the poor performance was primarily due to differences in the training data and labeling.

Our error analysis showed that retrained NegBERT and ALBERT made fewer errors than the other methods in predicting whether words occurred in speculative statements unrelated to abnormal findings. The number of all false-positive predictions by NegBERT and ALBERT was 4 and 5, respectively. Both were lower than the number of false-positive predictions made by NegEx and NegBio for this prediction task, indicating higher specificity. However, because we grouped all negative and speculative statements not related to abnormal findings into the same category, we could not calculate the exact value of specificity. Both models showed 100% sensitivity in identifying important diagnostic keywords in speculative statements unrelated to abnormal findings, with no false-negative predictions.

Owing to the complexity of BERT, we could not further analyze the causes of other errors.

## Discussion

### Principal Findings

#### Overview

This study found that 39.45% (4529/11,480) of the important diagnostic keywords occurred in negative or speculative statements unrelated to abnormal findings, posing a challenge for automatic labeling by LISs and information extraction techniques.

Our study proposes a deep learning method that accurately distinguishes whether diagnostic keywords are in negative or speculative statements unrelated to abnormal findings. Our research has revealed the shortcomings of existing methods, including NegEx, NegBio, and NegBERT, while highlighting the advantages of our proposed approach over these methods.

#### Limitation of NegEx and NegBio

We observed common errors in Spacy’s NegEx and NegBio that the expert rule adjustment could not resolve.

First, several vital errors in NegEx and NegBio, including errors related to trigger words in NegEx, tokenization errors in NegBio, and symbol-related errors in NegEx and NegBio, were attributed to interference from punctuation and numerals. For example, in the radiology reports in our sample, English sentences were often combined with numbers and punctuation marks and written as numbered or bulleted lists, such as “1.No evidence of aortic dissection” In addition, using symbols or abbreviations in the form of checklists was also common. For example, “Metastasis (−)” or “Anti-HCV [Negative]” were frequently used. Our results showed that NegEx and NegBio could not handle this issue correctly.

Second, NegEx and NegBio also caused many errors in the analyses where the simultaneous observation of multiple sentences is required. Our data showed that it is often necessary to examine multiple sentences simultaneously to determine whether speculative statements are associated with abnormal findings. For example, in “No CT evidence of large infarct. Suggest MRI to exclude hyperacute infarct if indicated,” without considering the first sentence, which denies the finding of infarct evidence, it cannot be determined that the “hyperacute infarct” in the second sentence is unrelated to the actual findings. NegEx and NegBio, which are designed to analyze sentences in isolation without considering contextual information, cannot meet this requirement.

Our results regarding NegEx are consistent with previous research of Wu et al [42], highlighting the importance of tuning algorithms such as NegEx to achieve optimal performance in different corpora. Our results also confirm that NegEx produces incorrect results owing to improper negation scope resolution [22].

We found that NegBio requires modifying expert-defined rules to improve its performance. Our study is the first to report NegBio’s limited generalizability in real-world radiology reports across all body parts. We also observed problems with the implementation of NegBio.

#### Limitation of NegBERT

Our experiment showed a significant improvement in NegBERT’s performance after retraining on our hospital data set. The difference in the training data and annotations is likely the reason for the initial poor performance of NegBERT.

This observation is consistent with previous findings that deep learning models such as BERT tend to perform poorly on out-of-domain corpora. For example, a study by Miller et al [39] using RoBERTa for negation detection on both in-domain and out-of-domain corpora observed *F*_1_-scores of 0.95 and 0.583, respectively. Our experiment supports this result and shows that the drop in *F*_1_-scores can be even worse depending on the corpus and task.

#### Advantages of ALBERT and BERT Transformer

We performed a comparison between the ALBERT and NegBERT methods and made the following key observations.

First, learning the negation cue and scope in 2 steps provides a limited performance improvement. Our method takes a different approach from NegBERT and traditional negation recognition studies in that our model learn the entire part of the sentence containing both the cue and scope in the same step without explicitly telling the model which word is the “cue” of the negation or speculation. However, the performance was still better than that of the retrained NegBERT. The study by Sergeeva et al [11] based on LSTM suggests that the deep learning method can learn negation cue information to some extent automatically, with performance comparable with that of automatic cue prediction algorithms. Our results show that BERT might have a similar capability. Our results suggest that providing additional cue information through expert annotation may not significantly improve performance compared with other factors, such as model selection, hyperparameter optimization, and training techniques.

Second, our results show that the model size and complexity do not necessarily correlate with improved performance. In our study, the fine-tuned ALBERT model outperformed larger and more complex models, including BERT and XLNet used by NegBERT, as well as RoBERTa used in the study by Miller et al [39]. The use of lightweight models, such as ALBERT, may have practical advantages, including reduced computational resource requirements and training time, compared with BERT [31].

In our study, ALBERT and retrained NegBERT outperformed NegEx and NegBio in terms of the number of false-positive predictions and specificity while maintaining 100% sensitivity in predicting whether keywords occurred in speculative sentences unrelated to abnormal findings. This task required multisentence context analysis of our data set, and our results suggest that BERT can look at multiple sentences simultaneously. The attention mechanism is a reasonable explanation for this phenomenon.

### Comparison With Prior Work

Our study fine-tuned the ALBERT model using a more comprehensive data set that included a broader range of imaging modalities and subspecialties than previous studies. Table 9 shows the best performances and corresponding data sets used in previous studies that detected whether named entities occurred in negation and speculation in radiology reports. The range of imaging modalities and subspecialties represented in the radiology reports in these studies was limited, such as chest x-ray reports only in the study by Peng et al [23] or brain CT and MRI reports only in the studies by Grivas et al [43] and Sykes et al [12]. We hypothesized that including a more diverse set of examination and imaging subspecialties in the data results in a more representative sample of the report content and improves the model’s generalizability. Our results support this hypothesis, as the ALBERT model showed only a 0.034 decrease in its *F*_1_-score on an unseen test data set with different disease types and inputs from different physicians.

Our experiments also address a more difficult speculation-detection task than previous studies; however, ALBERT still demonstrates good performance. This distinction requires the ability of the algorithm to consider multiple sentences simultaneously in our data set. To the best of our knowledge, our study is the first to propose a distinction between speculative sentences related and unrelated to abnormal findings based on the application scenario to facilitate more precise filtering and the first study to highlight the impact of the lack of multisentence analysis in negation detection algorithms.

**Table 9:** Comparison of best performances between studies distinguishing whether named entities occurred in negation or speculation.

Study	Algorithm	P^a^	R^b^	*F*_1_	Best^c^	N^d^	Task^e^	Type^f^
Our study	ALBERT^g^	0.991	0.992	0.991	Test data set	6000	ND^h^+S^*i^	All body parts
Sykes et al [12]	BiLSTM^j^	0.973	0.981	0.977	ESS^k^	630	ND+S^l^	Brain CT^m^ and MRI^n^
Peng et al [23]	NegBio	0.944	0.944	0.944	Chest x-ray	900	ND+S	Chest x-ray
Grivas et al [43]	Edie-R	0.925	0.943	0.934	ESS	630	ND+S	Brain CT and MRI

^a^P: precision.

^b^R: recall.

^c^Name of the best-performing data set. Other data sets are not included.

^d^Number of samples in the best-performing data set; other data sets are not included.

^e^Task performed in the study.

^f^Types of radiologic studies included in the study.

^g^ALBERT: A Lite Bidirectional Encoder Representations From Transformers.

^h^ND: negation detection.

^i^S^*^: detection of speculation unrelated to abnormal findings.

^j^BiLSTM: Bidirectional Long Short-Term Memory.

^k^ESS: Edinburgh Stroke Study.

^l^S: speculation detection.

^m^CT: computed tomography.

^n^MRI: magnetic resonance imaging.

### Implication in Clinical Practice

We found problems with NegEx and NegBio in that modifying expert-defined rules could not be solved, including difficulties with numbers and punctuation, implementation-specific challenges, and the design constraint of observing only a single sentence at a time; thus, NegEx and NegBio should be used cautiously or avoided in such situations to prevent errors. On the basis of our data, we also found that NegBio and NegBERT have limitations in generalizability, making them inappropriate for use without training or modeling.

Our results indicate that BERT is more suitable than NegEx and NegBio for tasks involving multisentence context analysis, similar to the experiment conducted in this study. NegEx and NegBio were designed for single-sentence analysis because they segmented the text into independent sentences. This approach limits the ability to incorporate contextual information from other sentences into the analysis. While NegEx and NegBio can perform binary classification of words in sentences as speculative or not, they lack the capacity for further granular differentiation based on contextual information.

We found that the training process of the transformers did not require 2 separate learning phases for cue and scope. Our findings could reduce the workload of expert annotation in clinical applications, as the explicit annotation of cues in a separate step requires additional work. This hypothesis needs further testing in future studies.

Our results show that deep learning models outperform non–deep learning methods, and lightweight models such as ALBERT can achieve superior performance and outperform other transformer-based models. However, fine-tuning based on the specific domain corpus and task is still essential regardless of the model used.

### Limitations

The data were obtained from 3 internal branches of a single institution and not from publicly available data sets. In addition, the speculation-detection task differed from previous studies in this area. The comparability of the performance with that of previous studies may be limited. If open data using the same annotation methodology become available, subsequent research could verify our findings by implementing the same model on the open data set.

Our study optimized the control methods (NegEx and NegBio), but we cannot exclude the possibility of further performance improvement by modifying or adding expert rules. However, this highlights the limitations of an expert rule–based approach, which requires experts not only to detect negations and speculations but also to summarize and modify rules manually. Moreover, expert rules cannot resolve the algorithmic design or implementation constraints.

To prevent the deep learning model from training failure, we combined negative statements with speculative statements unrelated to abnormal findings in the same category because of the low proportion of the latter. As a result, we cannot separately evaluate the model’s performance on negative and speculative sentences unrelated to abnormal findings or accurately quantify the latter’s performance. Nevertheless, metrics such as the number of false-positive predictions can still be used to compare the performance between methods.

### Conclusions

Manual free-text reporting remains the norm in radiology worldwide, hampering the ability to perform computer-assisted analyses. The presence of information irrelevant to the actual findings poses a significant challenge to the implementation of automatic radiology report highlighting, flagging, or information extraction.

Previous research on negation and speculation detection in radiology has aimed to identify all instances. Our study advances this by targeting only speculative statements unrelated to abnormal findings and improving the discrimination of relevant information using BERT’s multisentence contextual analysis capabilities.

Lightweight transformer models, such as ALBERT, can outperform NegEx, NegBio, and NegBERT on more complex and diverse real-world radiology reports. Despite achieving good results on public data sets, NegBio and NegBERT demonstrated different performances on more complicated real-world radiology reports.

Our research has potential applications in academia and clinical practice. Future studies may consider including lightweight models such as ALBERT. In clinical practice, our method achieved high performance. It can help algorithms such as keyword highlighting in hospital information systems to identify passages of potentially important information without false alarms, improving physician efficiency and health care quality. Our results also apply to radiology report information retrieval, such as search engines, in which negative and speculative statements unrelated to abnormalities can lead to incorrect results.

## Abbreviations

ALBERT: A Lite Bidirectional Encoder Representations From Transformers
BERT: Bidirectional Encoder Representations From Transformers
BiLSTM: Bidirectional Long Short-Term Memory
CRF: Conditional Random Field
CT: computed tomography
DeBERTa: Decoding-Enhanced Bidirectional Encoder Representations From Transformers With Disentangled Attention
DEEPEN: Dependency Parser Negation
DistilBERT: Distilled version of Bidirectional Encoder Representations From Transformers
ELECTRA: Efficiently Learning an Encoder That Classifies Token Replacements Accurately
ERNIE: Enhanced Representation through Knowledge Integration
LIS: laboratory information system
LSTM: Long Short-Term Memory
MRI: magnetic resonance imaging
RoBERTa: Robustly Optimized Bidirectional Encoder Representations From Transformers Pretraining Approach
